# Tandem Repeats Contribute to Coding Sequence Variation in Bumblebees (Hymenoptera: Apidae)

**DOI:** 10.1093/gbe/evy244

**Published:** 2018-11-06

**Authors:** Xiaomeng Zhao, Long Su, Sarah Schaack, Ben M Sadd, Cheng Sun

**Affiliations:** 1 Key Laboratory of Pollinating Insect Biology of the Ministry of Agriculture, Institute of Apicultural Research, Chinese Academy of Agricultural Sciences, Beijing, China; 2 Department of Biology, Reed College, Portland, Oregon, USA; 3 School of Biological Sciences, Illinois State University, Normal, Illinois, USA

**Keywords:** tandem repeats, genetic variation, adaptation, transcription regulation

## Abstract

Tandem repeats (TRs) are highly dynamic regions of the genome. Mutations at these loci represent a significant source of genetic variation and can facilitate rapid adaptation. Bumblebees are important pollinating insects occupying a wide range of habitats. However, to date, molecular mechanisms underlying the potential adaptation of bumblebees to diverse habitats are largely unknown. In the present study, we investigate how TRs contribute to genetic variation in bumblebees, thus potentially facilitating adaptation. We identified 26,595 TRs from the assembled 18 chromosome sequences of the buff-tailed bumblebee (*Bombus terrestris*), 66.7% of which reside in genic regions. We also compared TRs found in *B. terrestris* with those present in the assembled genome sequence of a congener, *B. impatiens*. We found that a total of 1,137 TRs were variable in length between the two sequenced bumblebee species, and further analysis reveals that 101 of them are located within coding regions. These 101 TRs are responsible for coding sequence variation and correspond to protein sequence length variation between the two bumblebee species. The variability of identified TRs in coding regions between bumblebees was confirmed by PCR amplification of a subset of loci. Functional classification of bumblebee genes where coding sequences include variable-length TRs suggests that a majority of genes (87%) that could be assigned to a protein class are related to transcriptional regulation. Our results show that TRs contribute to coding sequence variation in bumblebees, and thus may facilitate the adaptation of bumblebees through diversifying proteins involved in controlling gene expression.

## Introduction

Tandem repeats (TRs) are DNA tracts in which a short DNA sequence, dubbed a repeat unit, is repeated several times in tandem and they are ubiquitous in the genomes of diverse species (Vinces et al. 2009; Gemayel et al. 2010; Melters et al. 2013). Most mutations in TRs are due to the variation in repeat unit number that occurs when one or more repeat units are added or deleted via a variety of different mutational mechanisms (e.g., DNA polymerase slippage; Tachida and Iizuka 1992). Because they are known to be highly variable, TRs are also known as VNTRs (variable number of TRs; Gemayel et al. 2010). A number of local cellular processes (e.g., DNA replication, recombination, and faulty DNA repair) and other factors (e.g., DNA damage, oxidative stress due to metabolism) are known to cause mutations in TRs, thus the frequency of mutations at these loci is thought to be 100–10,000 times higher than point mutations (Tachida and Iizuka 1992; Paques et al. 1998; Schmidt and Mitter 2004; Rando and Verstrepen 2007; López et al. 2010).

Mutations in TRs can have phenotypic consequences. Firstly, mutations in TRs residing in coding regions can impact the size, structure, function, or processing of messenger RNAs or proteins. Several neurodegenerative diseases have been linked to the repeat unit number variation of TRs located in coding regions, the most famous case being the abnormal expansion of a CAG repeat in exon 1 of the *IT15* gene leading to Huntington’s disease (HD). In this instance, repeat unit numbers ranging from 6 to 35 are found in healthy individuals, whereas alleles with 40 repeats or more cause HD (Duyao et al. 1993; Gatchel and Zoghbi 2005).

In addition to their role in disease, TRs in coding regions can also confer adaptive phenotypic variability (reviewed in Kashi and King 2006). The repeat unit number variation in TRs located in the *FLO1* gene in *Saccharomyces cerevisiae* generates plasticity in cell adhesion to substrates (Verstrepen et al. 2005). In canines, variable TRs located in developmental genes confer variability to skeletal morphology (Fondon and Garner 2004). Further, mutations in TRs located in noncoding regions can also have significant effects. Variable-length TRs have been shown to influence transcription factor binding, as well as potentially changing DNA structure, packaging, and spatial dynamics, in addition to changing the secondary structure of RNA molecules once transcribed. TRs in promoters change gene expression in yeast (Vinces et al. 2009), and contribute to gene expression variation in humans (Gymrek et al. 2016). Therefore, given that TRs are highly mutable regions in the genome, and thus represent a significant source of variation, in cases where this variation is at loci influencing morphological, physiological, and behavioral traits, it could facilitate adaptation to different environments (Tautz et al. 1986; Verstrepen et al. 2005; Fidalgo et al. 2006; Vinces et al. 2009; Gemayel et al. 2010; Fonville et al. 2011; Zhou et al. 2014; Feliciello et al. 2015; Xu et al. 2017).

Bumblebees (Hymenoptera: Apidae) are a genus of pollinating insects that play an important role in crop production and natural ecosystem services (Fontaine et al. 2006; Velthuis and van Doorn 2006; Garibaldi et al. 2013). They are distributed widely across the globe, from Greenland to the Amazon Basin and from sea level to altitudes of 5,800 m in the Himalayas (Williams 1985). Bumblebees occupy a remarkably wide diversity of habitats, from alpine meadows to lowland tropical forest (Sakagami 1976). However, to date, molecular mechanisms underlying the adaptation of bumblebees to such a diverse array of habitats are largely unknown. Genetic variation is important for adaptation to new environments (Lande and Shannon 1996; Barrett and Schluter 2008; Paaby and Rockman 2014), however, little is known about sources or levels of genetic variation in bumblebees (but see Lozier et al. 2011; Maebe et al. 2016).

In the present study, we performed a systematic examination of TRs in the bumblebee genome and investigate their contribution to genetic variation in this taxon. We further examine the potential functional significance of the genetic variation introduced by TRs to bumblebee genes, specifically. Lastly, we discuss the potential significance of the genetic variation, especially as it may influence the regulation of gene expression, by comparing the levels of variation observed within and between species.

## Materials and Methods

### Genomic Sequences, Annotation, and Predicted Proteins

The genomic sequences, genome annotation, and predicted protein sequences of *Bombus terrestris* were downloaded from GenBank (https://www.ncbi.nlm.nih.gov/genome/2739, last accessed on April 5, 2016; GenBank assembly accession of GCF_000214255.1 [Bter_1.0]). The genomic sequences and predicted protein sequences of *Bombus impatiens* were downloaded from GenBank (http://www.ncbi.nlm.nih.gov/genome/3415, last accessed on April 5, 2016; GenBank assembly accession of GCA_000188095.2 [BIMP_2.0]). The genomic sequences of honeybee (*Apis mellifera*) were downloaded from GenBank (http://www.ncbi.nlm.nih.gov/assembly/GCF_000002195.4/, last accessed on September 8, 2018; GenBank assembly accession of GCA_000002195.1 [Amel_4.5]).

### Bumblebee Genomic DNA

The three worker specimens of *Bombus terrestris* were collected in the summer of 2017 in Burqin County, Xinjiang Uygur Autonomous Region, China from three different sites within a 6-km range of a previously collected conspecific (GPS coordinates: latitude 48.19179; longitude 87.02355). The species identity was confirmed by DNA barcoding of all *B. terrestris* specimens, with sequences being identical to those of previously sequenced specimens of *B. terrestris* from this region (NCBI accession number: GU085204.1). Each of five males of *Bombus impatiens* was sourced from a distinct laboratory raised colony, which had been founded by field caught queens collected in Central Illinois, United States (GPS coordinates: latitude 40.657011; longitude −88.873755), in the spring of 2017. DNA was extracted from each bumblebee specimen using the Blood & Cell Culture DNA Mini Kit (Qiagen).

### Identification of TRs in the *B. terrestris* Genome

Each of the 18 chromosome sequences of *B. terrestris* was uploaded to the Tandem Repeats Database (TRDB) (Gelfand et al. 2007). First, the sequence of each chromosome was analyzed using Tandem Repeats Finder (TRF) using default parameters: 2, 7, 7, 50 (match, mismatch, indels, minimal alignment score) (Benson 1999). As the bumblebee genome is AT-rich (∼63%), poly A/T or AT/TA dinucleotides can occur by chance. Thus, to decrease the false positive rate of TR identification, TRs with repeat unit lengths of less than two or array lengths of less than 30 bp were discarded. Finally, redundant TRs reported for the same loci were excluded using the Redundancy Elimination tool at TRDB. For redundancy elimination, if TRs overlapped by >50% of their length, the repeat with the longer array was retained, or in the case of ties, the repeat with the shorter repeat unit length was retained. Manual correction was carried out when necessary.

### Evaluation of the Completeness of TRs and Protein-Coding Genes in the Assembled Genome of *B. terrestris*

To understand how many TRs may be missed when mining TRs from the assembled genome sequence, we downloaded the eleven 454 GS FLX Titanium runs of *B. terrestris* WGS data from NCBI (https://www.ncbi.nlm.nih.gov/sra/SRX016989). After getting rid of potential sequencing artifacts using locally installed cd-hit-454 (https://github.com/weizhongli/cdhit), we searched the remaining 454 reads for TRs using TRF software (parameters and thresholds were the same as used in assembled genome). Considering the sequencing depth of the assembled *B. terrestris* genome was 21× (Sadd et al. 2015), we divided the total number of TRs estimated from WGS reads by 21. Also, we used gsMapper implemented in NewblerDataAnalysis_2.9 (https://contig.wordpress.com) to map 454 shotgun reads that contain TRs back to the assembled *B. terrestris* genome (command: runMapping -ml 80 -mi 95) to evaluate to what extent we underestimate the total number of TRs when mining TRs from assembled genome sequence.

To understand if, like TRs, protein-coding genes are also missed from the genome assembly of *B. terrestris*, we used the software BUSCO 3.0.2 (https://busco.ezlab.org/) to evaluate the annotation results of *B. terrestris* genome (predicted proteins), with the use of hymenoptera_odb9 data set.

### Characterizing the Molecular Features of TRs

The molecular features of TRs in the assembled genome of *B. terrestris*, including repeat unit and repeat unit length distribution, TR array length distribution, and genomic locations, were analyzed using the set of nonredundant TRs obtained from the above step and a set of in-house Perl scripts, which are available at GitHub (https://github.com/suncheng781120/Tandem-repeat-analysis).

### Mining Variable-Length TRs between *B. terrestris* and *B. impatiens*

The sequence of each TR array, along with 100 bp of upstream and downstream flanking sequence, was extracted from the soft-masked *B. terrestris* genomic sequences (GCF_000214255.1**)**. If there were continuous lower-case letters longer than 10 bp in either flanking sequence, indicating that the TR may reside in a repetitive region, the TR locus was excluded from further analysis. The sequences of the remaining TR loci, along with their 100-bp flanking regions, were used as queries in BLASTn searches against the genomic sequence of *B. impatiens*, with an e-value cutoff of 1e-10. For each query, we retained the best hit (based on e-value) that included both the TR array sequence and >95 bp of flanking sequences on both sides (because these hits likely represent the query’s orthologous locus in the *B. impatiens* genome). Finally, the pairwise alignments between the sequences of the TR arrays in *B. terrestris* and their best hits in *B. impatiens* were parsed to check if sequence length variation was observed within the TR array.

### Identification of TRs Contributing to Coding Sequence Variation

The coordinates of the identified variable-length TRs from the above step were used to search against the genome annotation of *B. terrestris* (downloaded from GenBank, see above) to identify those that resided in predicted coding DNA sequence (CDS). Then, whenever one variable-length TR was found in the coding sequence of one *B. terrestris* gene, the full-length protein sequence encoded by this *B. terrestris* gene was used as a query in a BLASTp search against the protein database of *B. impatiens* to find the best hit from *B. impatiens*. Finally, based on the pairwise alignments between the protein sequences of the query and its best hit, we checked for amino acid sequence variation caused by the variable-length TR (e.g., if one or more amino acid residues were added or deleted from one of the bumblebee species). If there was variation in the amino acid sequence, the variable-length TR was considered to contribute to bumblebee coding sequence variation.

### PCR Amplification of Identified Variable-Length TRs in Coding Sequences

The sequences of identified variable-length TRs residing in coding sequences, along with 200 bp of flanking sequences, were extracted from the genomic sequence of *B. terrestris*, and PCR primers were designed using Primer 3 (Untergasser et al. 2012). Then, with primers spanning the variable-length TRs, PCR was used to amplify genomic DNA samples extracted from *B. terrestris* and *B. impatiens* specimens (detailed PCR primer information is available in supplementary file 1, Supplementary Material online).

A 15-μl reaction mixture composed of 50 ng of template DNA, 0.3 µl of 10 mM each deoxynucleotide triphosphate (dNTP), 0.4 units of *Taq* DNA polymerase (Sangon Biotech, Shanghai, China), 1.5 µl of 10× PCR buffer with Mg^2+^, and 1.2 µl of 10 µmol/l forward and reverse PCR primers was prepared. Amplification was carried out using the following reaction conditions: initial denaturation at 94 °C for 5 min, followed by 35 cycles of 30 s at 94 °C, 30 s at 56 °C, and 30 s at 72 °C, with a final extension at 72 °C for 10 min. About 3 μl of PCR products were separated on 8% polyacrylamide denaturing gels, and the bands were revealed by silver-staining (Panaud et al. 1996).

### Comparative Genomics of Variable-Length TRs in Coding Sequences across Species

The sequences of each variable-length TR array, along with their 100 bp of flanking sequences, were extracted from the *B. terrestris* genomic sequences and were used as queries in BLASTn searches against the genomic sequence of *B. impatiens* and *Apis mellifera*, with an e-value cutoff of 1e-5. For each query, we retained the best hit (based on e-value) from *B. impatiens* and *A. mellifera* that included both the TR array sequence and >90 bp of flanking sequences on both sides. Where eligible hits were found in both *B. impatiens* and *A. mellifera*, Clustal Omega (https://www.ebi.ac.uk/Tools/msa/clustalo/) was used to align the query sequence from *B. terrestris* and their best hits from *B. impatiens* and *A. mellifera* to provide information on the macroevolutionary changes of the focal TR arrays. Manual correction was carried out when necessary.

### Functional Classification of Genes Containing Variable TRs

We used the predicted protein sequences of *B. terrestris* genes containing variable-length TRs as queries to do local BLASTp against the downloaded Swiss-Prot database (http://www.uniprot.org/uniprot/, last accessed on September 1, 2016), with an e-value cutoff of 1e-10. The UniProt accession of the best hit was used to represent this gene. The collected UniProt accessions were uploaded onto the PANTHER server (http://pantherdb.org/) and classified by the PANTHER system (Mi et al. 2013). If a TR-containing gene did not get a significant hit in the Swiss-Prot database or the obtained UniProt accession could not be mapped using PANTHER, we used the protein sequence encoded by the *B. terrestris* gene as query to search against the PANTHER library Version 12.0 (http://pantherdb.org/) with default settings to get a UniProt accession, which could be recognized by the PANTHER system, to represent the *B. terrestris* gene.

## Results

### The Identification of TRs in Bumblebee Genome

Using the 18 chromosome sequences of *B. terrestris* (Sadd et al. 2015) as a reference, we identified 26,595 TRs in the bumblebee genome (after redundancy elimination; see Materials and Methods). Because TRs, especially centromeric and subtelomeric TR arrays, are notoriously tough to assemble, they are frequently absent from a genome assembly (Treangen and Salzberg 2011; Miga 2015). To understand how many TRs might have been missed when mining TRs from the genome assembly of *B. terrestris*, we used two methods: 1) identify TRs directly from the whole genome shotgun (WGS) reads, and 2) mapping WGS reads that contain TRs back to the assembled genome of *B. terrestris*. Results indicated that ∼94,758 TRs could be identified using the first method, which is much higher than the reported number of TRs (26,595) when mining TRs from the 18 assembled chromosome sequences. Searching TRs from WGS reads, however, could yield an inflated TR estimate (e.g., if one TR spans two WGS reads, it could be identified as two TRs). When mapping WGS reads back to the genome assembly of *B. terrestris* (method 2), only 46.8% of TR-containing WGS reads could be mapped to the assembled genome, indicating that over half of the TR-containing reads failed to be assembled into the reference genome sequence. Thus, our estimate of ∼25,000 TRs represents a lower bound for the total number of TR arrays in the genome, and likely misses at least 50% of the TRs that might occur in regions that are difficult to assemble.

To understand if, like TRs, protein-coding genes are also missed from the genome assembly of *B. terrestris*, we used the software BUSCO to evaluate the assembled *B. terrestris* genome. Our results indicate that 4,403 of the 4,415 (99.7%) Hymenoptera-wide single copy genes can be identified in the assembled genome, and thus that protein-coding genes were well assembled in the reference genome sequence of *B. terrestris*. Therefore, as found in other species (Treangen and Salzberg 2011; Miga 2015), while some TRs, especially those reside within highly repetitive centromeric or subtelomeric regions, will be missed from assembled genome of *B. terrestris*, those occurring in the protein-coding genes should be readily identifiable. Because the main purpose of this study is to understand the contribution of TRs to the coding sequence variation of protein-coding genes, TRs in centromeric and subtelomeric regions (which are generally gene-poor) are missed, we expect it to have little effect on the main goal of our present study.

### Molecular Features of TRs in the Bumblebee Genome

**Fig. 1.**
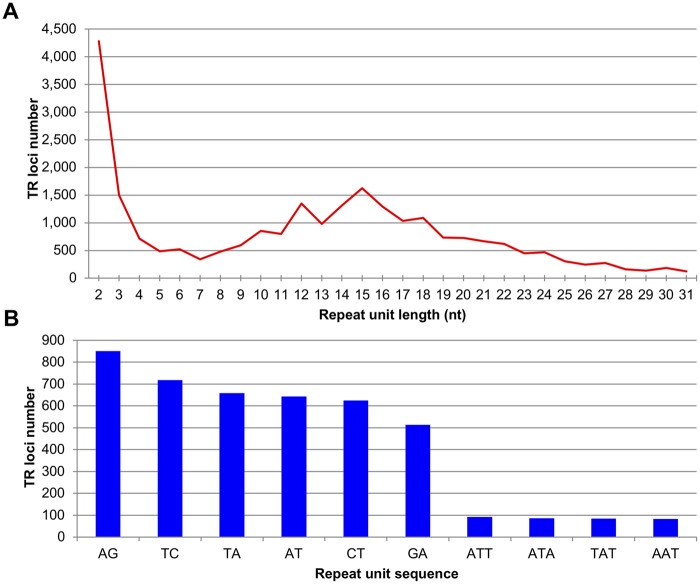
—Repeat unit features for identified bumblebee TRs. (*A*) Repeat unit length distribution of TRs. Only repeat unit lengths, at which there are >100 TR loci, are shown. (*B*) The top ten most abundant repeat unit sequences.

The distribution of repeat unit lengths of TRs in the assembled genome of *B. terrestris* is summarized in figure 1*A*. In general, the number of TR loci detected decreases with increasing repeat unit length. However, there are exceptions: two peaks occur at repeat unit lengths of 12 and 15 nt. The top ten most abundant repeat unit sequences, all either dinucleotide or trinucleotide, were quantified (fig. 1*B*), with the repeat unit “AG” being the most abundant in the assembled genome.

**Fig. 2.**
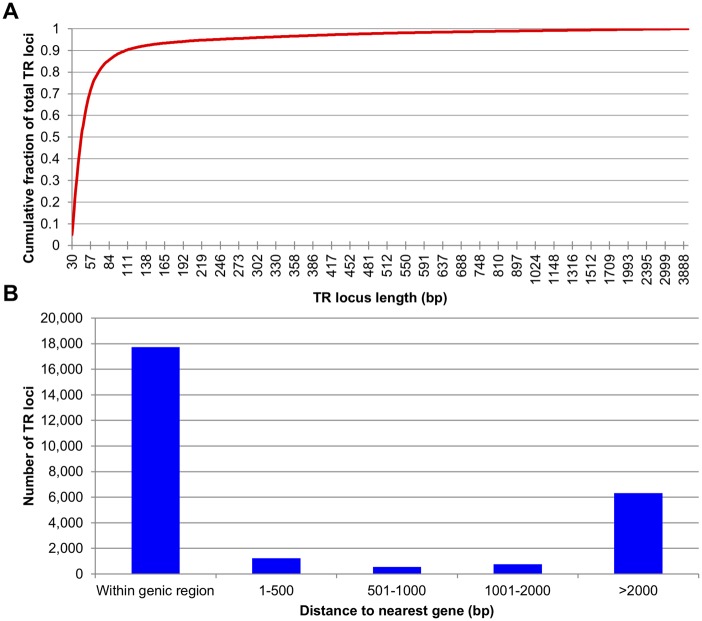
—Distribution features for bumblebee TR loci. (*A*) TR locus length distribution. (*B*) The distance between TRs and predicted genes. As shown in the figure, a majority of TRs identified from assembled portion of *Bombus terrestris* genome reside within genes.

Most of the TR loci in the assembled genome of *B. terrestris* are relatively short, with 90% of TR loci having a length that is equal to or shorter than 111 base pairs (bps) (fig. 2*A*). To characterize the genome-wide distribution of TRs, the coordinates of TR loci were compared with the genome annotation for *B. terrestris*. Our results indicate that 66.7% (17,739 out of 26,595) of TRs identified from the assembled bumblebee genome were located within predicted genes (fig. 2*B*).

### TRs Contribute to Genetic Variation in Bumblebees

To understand the contribution of TRs to genetic variation in bumblebees, TRs identified in the nonrepetitive regions of the *B. terrestris* genome were used as queries to find their orthologous loci in another sequenced bumblebee genome, *B. impatiens*. Based on the pairwise alignments between the TR array sequences from the two bumblebee species, we identified variable TRs between them (see Materials and Methods). A total of 2,862 TRs were located within the nonrepetitive regions of the *B. terrestris* genome, and, relative to *B. impatiens*, 1,137 of them are variable-length TRs (supplementary file 2, Supplementary Material online).

**Fig. 3.**
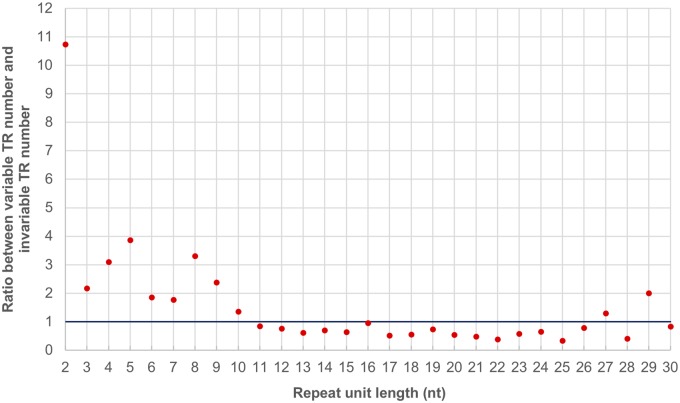
—The relationship between repeat unit length and observed mutation probability for bumblebee TRs. The ratio between the number of TRs showing length variability and the number of TRs that do not exhibit variability in length was plotted against the repeat unit length of TRs.

To understand if there are certain repeat unit lengths of TRs that are most likely to be array size (repeat unit number) variable between the two bumblebees, we calculated the ratio between the number of TRs showing variability in length between the two species and the number of TRs that do not exhibit variability in length for each repeat unit length, and plotted the ratio against the repeat unit length of TRs (fig. 3). Generally, TRs with repeat unit lengths ranging from 2 to 10 bp are more likely to be array size (repeat unit number) variable than TRs with longer repeat units (fig. 3).

### TRs Contribute to Coding Sequence Variation in Bumblebees

To identify TRs generating coding sequence variation in bumblebees, we compared the genomic coordinates of the 1,137 variable TRs identified from the above step with those annotated as coding sequence (CDS) in the *B. terrestris* genome. We constructed pairwise alignments between protein sequences containing variable-length TRs to identify TRs generating protein sequence length variation between the two bumblebee species (see Materials and Methods). Based on this analysis, 101 of the 1,137 variable TRs were responsible for coding sequence variation (supplementary file 3, Supplementary Material online) and corresponded to protein sequence length variation (supplementary file 4, Supplementary Material online).

**Fig. 4.**
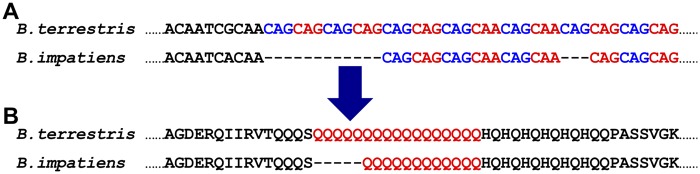
—An example of TRs contributing to bumblebee coding sequence variation. (*A*) Pairwise alignments of TR arrays between *Bombus terrestris* and *B. impatiens*. Colored letters indicate TR array sequences, while black letters show their flanking sequences. The TR array has a repeat unit of CAG, and there are five more repeat units in *B. terrestris* than in *B. impatiens*. The coordinate for the variable TR is NC_015770.1: 2190704–2190753 in *B. terrestris*. (*B*) Pairwise alignments of protein sequences encoded by genes containing the variable TR. Colored letters indicate TR array sequences, while black letters show their flanking sequences. There are five more glutamine residues (Q) in *B. terrestris* than in *B. impatiens*. Genes containing this variable TR encode nuclear receptor corepressor (protein IDs are XP_012166765.1 and XP_012249688.1 in *B. terrestris* and *B. impatiens*, respectively).

In figure 4, we show one example of a TR generating coding sequence variation; the focal TR, which resides within a gene encoding a nuclear receptor corepressor, has a repeat unit of CAG (which encodes glutamine). From the DNA sequence alignments (fig. 4*A*), we can see that the sequence divergence between the two bumblebees was caused by differential gain/loss of the repeat unit “CAG.” There are five more repeat units in *B. terrestris* than in *B. impatiens* (fig. 4*A*). As a result, there are five more glutamine residues (represented by Q in the one-letter code) in the protein sequence encoded by the TR-containing gene in *B. terrestris* than in *B. impatiens* (fig. 4*B*).

**Fig. 5.**
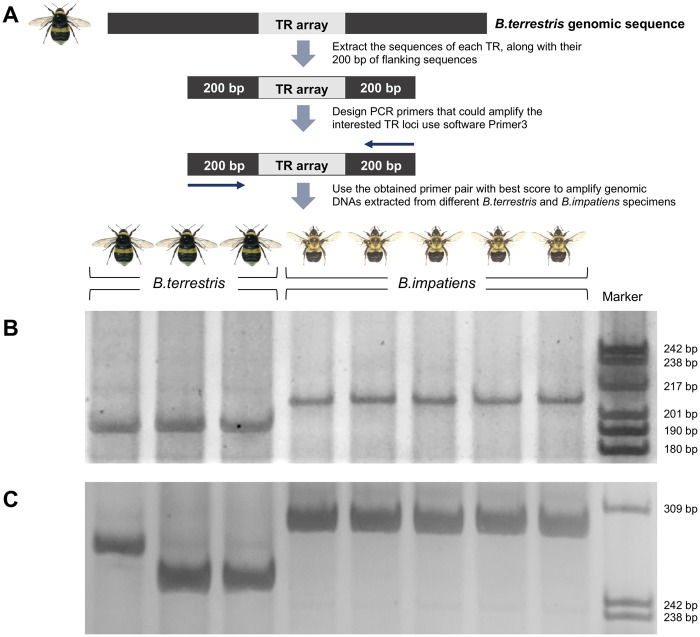
—PCR amplification of variable-length TRs residing in coding sequences in specimens of *Bombus terrestris* and *B. impatiens*. (*A*) A schematic showing the principle of primer design and PCR amplification. (*B*) PCR amplification of the variable-length TRs residing in the gene that encodes FERM, RhoGEF, and pleckstrin domain-containing protein (protein ID: XP_012169724.1 for *B. terrestris*). This figure indicates that, for the given TR locus, there is fixed length variation between the two species. (*C*) PCR amplification of the variable-length TRs residing in the gene that encodes hexamerin (protein ID: XP_012169664.1 for *B. terrestris*). This figure indicates that, for the given TR locus, there is length variation within species.

To further confirm that TRs contribute to coding sequence variation in bumblebees, we designed PCR primers that span the identified variable TRs in coding sequences and used them to amplify the genomic DNA extracted from three unrelated specimens of *B. terrestris* and five unrelated specimens of *B. impatiens* (fig. 5*A*). Our results (summarized in table 1, with details available in supplementary file 1, Supplementary Material online) indicate that 19 of the 29 TR loci amplified exhibit interspecific length variation between *B. terrestris* and *B. impatiens*, with no length variation within species (denoted as Fixed variation). Eight of the 29 TR loci showed intraspecific length variation within at least one species, but the distributions of lengths in the two species do not overlap (denoted as Variation within species). Two of the 29 TR loci show transspecies variation, with overlapping distributions of length in the two species (denoted as Not fixed). Examples of the PCR amplification results revealing inter- and intraspecific variation of TRs in coding sequences can be seen in figure 5*B* and *C*, respectively. Altogether, our results suggest that TRs contribute to coding sequence variation in bumblebees.

**Table 1:** The Summary for the PCR Amplification of TR Loci in Coding Sequences

Total Loci #	Successfully Amplified #	Fixed Variation #	Variation within Species #	Not Fixed #
30	29	19	8	2

Note.—Detailed results are available in supplementary file 1, Supplementary Material online.

We further examined the repeat unit length of the 101 variable-length TRs found in coding sequences. We observed 35 of them have a repeat unit length of 3, with all the other variable TRs having a repeat unit length of multiples of three (supplementary file 5, Supplementary Material online). This finding is consistent with previous research in other species, which indicates that selection should favor or tolerate mutations that avoid high impact frameshift mutations (Young et al. 2000; Richard and Dujon 2006; Legendre et al. 2007; Mularoni et al. 2010).

### Comparative Genomics of Variable-Length TRs in Coding Sequences

**Fig. 6.**
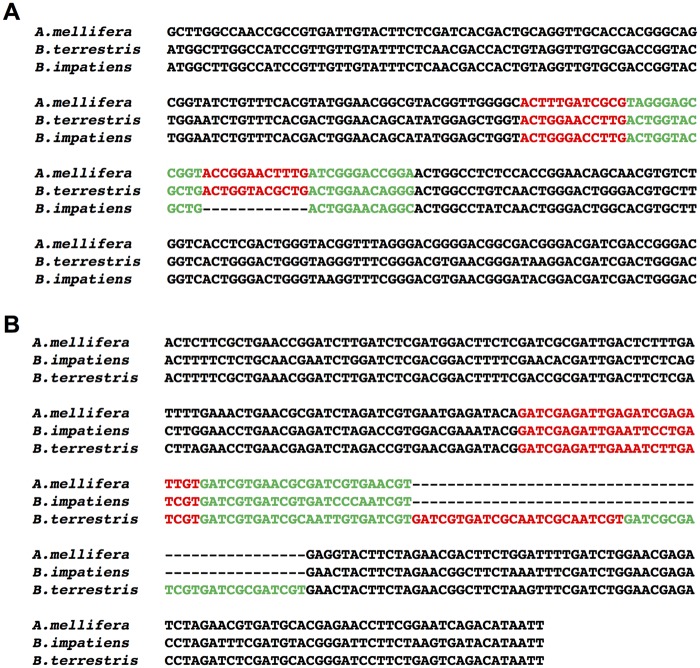
—Comparison of variable-length TRs in coding sequences across species. (*A*) TR evolved by the loss of one repeat unit in *Bombus impatiens*. Colored letters indicate the repeat units of focal TR array, while black letters show its flanking sequences. The sequences involved in the multiple alignments are *Apis mellifera* (Group9: 2931618–2931857), *B. terrestris* (NC_015770.1: 10495478–10495717), and *B. impatiens* (NT_176565.1: 370528–370755). The gene containing this variable TR encodes a cyclin-dependent kinase inhibitor (protein ID is XP_012167390.1 in *B.* t*errestris*). (*B*) TR evolved by the gain of two repeat units in *B. terrestris*. Colored letters indicate the repeat units of focal TR array, while black letters show its flanking sequences. The sequences involved in the multiple alignments are *A. mellifera* (Group2: 3483900–3484134), *B. terrestris* (NC_015763.1: 9110312–9110594), and *B. impatiens* (NT_176945.1: 210140–210374). The gene containing this variable TR encodes an RNA polymerase-associated protein (protein ID is XP_012175579.1 in *B. terrestris*).

Of the 101 variable TRs between the two bumblebees, 33 of them could be matched to orthologs in the honeybee (*Apis mellifera*) genome. For 27 of the 33 TR loci, the lengths of TR arrays in the two bumblebees are both different from that of honeybee. For the remaining 6 of the 33 TR loci, the lengths of TR arrays in one of the two bumblebees are the same as that of honeybee, which could be indicative of the ancestral state of the TR loci, and thus be used to infer the evolutionary trajectories of these TR arrays in bumblebees. Of the six TR loci, four show divergence from the putative ancestral state in *B. terrestris*, while two exhibit divergence in *B. impatiens*. We show two examples of such TR loci in figure 6. The variable TR locus shown in figure 6*A* resides within a gene encoding a cyclin-dependent kinase inhibitor (protein ID: XP_012167390.1), and this TR locus exhibits a loss of one repeat unit in *B. impatiens* (fig. 6*A*). The variable TR locus shown in figure 6*B* resides within a gene encoding a RNA polymerase-associated protein (protein ID: XP_012175579.1), and this TR locus exhibits a gain of two repeat units in *B. terrestris* (fig. 6*B*) relative to the other two species.

Taken together, these results suggest that TR arrays in bumblebees could evolve by differential loss or gain of one or more repeat units. Moreover, when we verified the variability of TRs in coding regions between bumblebees by PCR amplification of a subset of variable TR loci (as shown in fig. 5), the two TR loci shown in figure 6 were included. From the PCR amplification results (supplementary file 1, Supplementary Material online), we found that the length of PCR-amplified products from the two TR loci exhibit interspecific length variation between *B. terrestris* and *B. impatiens*, with no length variation within species. That is, the lengths of two TR loci are the same among the three independently collected specimens of *B. terrestris* and among the five independent specimens of *B. impatiens*, but the lengths between *B. terrestris* specimens and *B. impatiens* specimens are different. This indicates that the two TR loci may be under selection and thus have the potential to be involved in the adaptation of bumblebees. In addition to these two variable TR loci that have recognizable orthologs in honeybee genome, there are 17 more TR loci that exhibit interspecific length variation between *B. terrestris* and *B. impatiens*, with no length variation within species (table 1). These TR loci could also be under selection and therefore functionally important for bumblebees.

### Protein-Coding Gene Sequence Variation Driven by TRs in Bumblebees

**Fig. 7.**
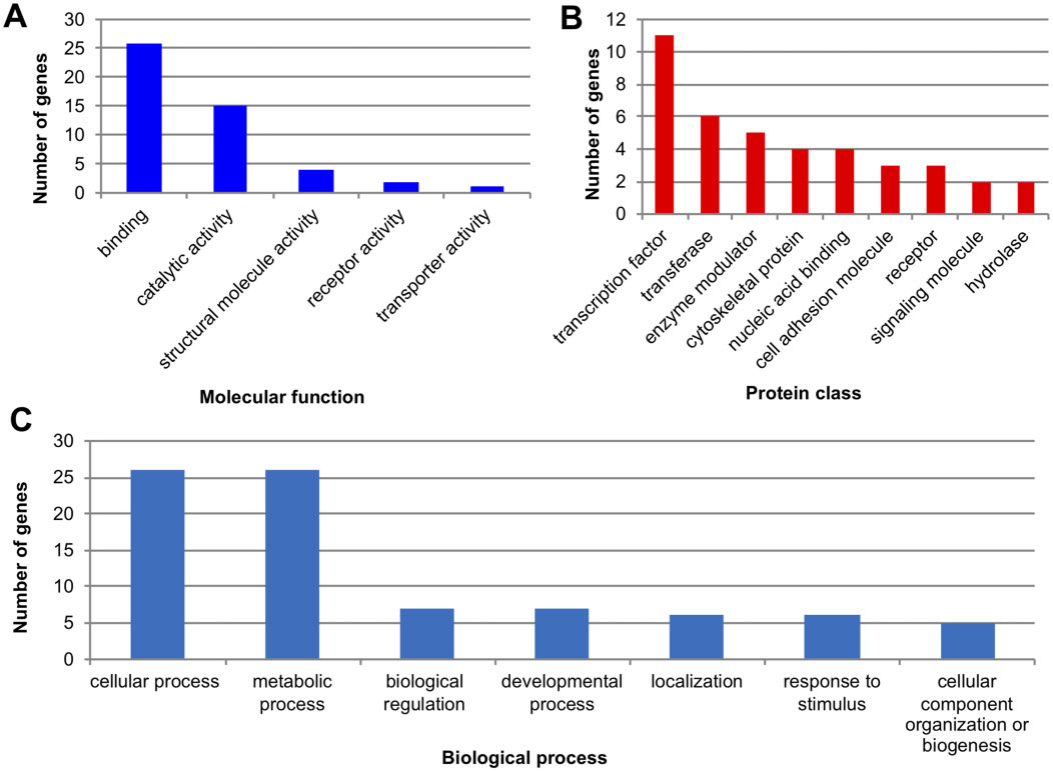
—Functional classification of genes that include variable-length TRs. (*A*) The number of genes classified in each molecular function category. (*B*) The number of genes classified in each protein class. The gene number shown in the nucleic acid binding category excludes transcription factors. (*C*) Biological processes that genes including variable-length TRs are involved in.

The identified 101 variable-length TRs that contribute to coding sequence variation in the sequenced bumblebees are found in 85 protein-coding genes (some of the genes have more than one variable TRs in them). We performed a functional classification using PANTHER, from which 74 of the genes could be functionally classified. Over half of the classified genes (26 out of the 48 genes that could be assigned a molecular function) are involved in binding, which is defined as the selective, noncovalent, often stoichiometric, interaction of a molecule with one or more specific sites on another molecule (fig. 7*A*). The second most frequent molecular function is catalytic activity, with 15 genes falling in this category. Other molecular functions of classified genes include structural molecular activity, receptor activity, and transporter activity (fig. 7*A*).

Proteins encoded by those genes containing variable-length TRs were assigned to 18 protein class categories, and the top nine categories (categories having two or more genes) are shown in figure 7*B*. The most frequent protein class category represented is transcription factors, and a total of 11 genes were found to encode them (fig. 7*B*). Based on the available databases that curate transcription factors in the genome of *B. terrestris*, the KEGG BRITE database (http://www.genome.jp/kegg/brite.html) identifies 271 genes as eukaryotic transcription factors (last updated on June 8, 2018), and the Regulator database (http://www.bioinformatics.org/regulator) returns 529 metazoan transcription factors (including 76 potential transcription factors, last accessed on July 5, 2018). Thus, based on the current assignment of the Regulator database, there are 529 transcription factors in *B. terrestris* among the ∼13,000 protein-coding genes in the genome (Kapheim et al. 2015). While 4.06% (529 out of 13,000) of bumblebee genes encode transcription factors, 12.94% (11 out of 85) of the classified genes containing variable-length TRs are transcription factors, which represents a >3-fold overrepresentation in this category. Other identified protein class categories include transferase and enzyme modulators (fig. 7*B*).

Bumblebee genes where coding sequences contained variable-length TRs are involved in a variety of biological processes (fig. 7*C*). The most frequent biological process categories are cellular and metabolic processes, each with 26 classified genes. Other biological processes represented include biological regulation, developmental processes, and response to stimulus. Genes containing variable-length TRs were involved in eight known pathways, namely, *Wnt* signaling, Nicotinic acetylcholine receptor signaling pathway, Apoptosis signaling pathway, Alzheimer disease-presenilin pathway, 5HT2 type receptor mediated signaling, p38 MAPK pathway, Heterotrimeric G-protein signaling pathway, and Huntington’s disease pathway. Interestingly, one bumblebee gene where the coding sequence contains variable-length TRs has the same trinucleotide repeat expansion (CAG) as that which causes Huntington’s disease in humans (fig. 4), and was determined to be associated with the Huntington’s disease pathway by PANTHER.

## Discussion

Genomic TRs are widespread in diverse species, where they represent highly dynamic regions of mutation, which can facilitate the rapid evolution of coding and regulatory sequences (Gemayel et al. 2010). However, to date, little is known about TRs in bumblebees, despite their importance as pollinator species and their wide range of habitats (Sakagami 1976; Williams 1985). The present study represents the first systematic analysis of TRs in bumblebees. Based on our search criteria, there are over 26,000 TRs in the assembled genome of *B. terrestris*, and 1,137 of which are polymorphic when compared with a closely related species, *B. impatiens*. Our TR identification method will underestimate the true number of TRs in bumblebees (see Results). In addition, variable-length TRs were identified by using TRs with nonrepetitive flanking sequences only (see Materials and Methods) and were based on a comparison of only 2 out of the 250 known bumblebee species (Cameron et al. 2007; Hines 2008). Therefore, our results represent a conservative estimate of the effect of TRs on bumblebee genetic variation, and the true amount of sequence variation contributed by TRs is likely much greater.

Because genetic variation is an essential starting point for adaptation to new environments (Lande and Shannon 1996; Barrett and Schluter 2008; Paaby and Rockman 2014), we postulate TRs may contribute to adaptation of bumblebees across the many niches in which they are found. With threats to bumblebees of upmost concern given recent population declines (Cameron et al. 2011), TRs may also determine susceptibility and evolutionary responses to proposed environmental stresses (Goulson et al. 2015).

Interestingly, in this study, we find evidence for changes in protein-coding sequence due to variation in TRs, and the frequency of such changes are most frequently observed in proteins known to influence gene expression. Both changes in protein sequences and changes in gene expression could drive adaptation, although the relative importance of these two molecular mechanisms has long been controversial (King and Wilson 1975; Fondon and Garner 2004; Wray 2007; Hancock et al. 2011; Fraser 2013). To understand the possible molecular mechanisms facilitating adaptation in bumblebees through TRs, we focus on changes in protein sequences rather than changes in gene expression, because even *cis*-regulatory sequences, which are directly related to changes in gene expression (Wray 2007), have not been extensively annotated yet in bumblebee genomes.

In this study, we searched for TRs that generate coding sequence variation, which in turn produce proteins of varying lengths (supplementary file 4, Supplementary Material online). In terms of the protein-coding changes we observed, for the 101 variable-length TRs identified, all the repeat units have a length of multiples of three (supplementary file 5, Supplementary Material online), which is consistent with findings in other species suggesting that natural selection may favor mutations that avoid frame-shifts (Young et al. 2000; Richard and Dujon 2006; Legendre et al. 2007; Mularoni et al. 2010). Mutations in TRs altering the length of protein sequences without introducing frame-shifts have the potential to majorly increase the functional diversity of host genes (Fondon and Garner 2004; Caburet et al. 2005; Verstrepen et al. 2005; Gemayel et al. 2010; Radó-Trilla et al. 2015). Our functional classification, however, further revealed that the most frequent protein class category exhibiting variable-length TRs is transcription factors, with a total of 11 genes (fig. 7*B*, supplementary file 5, Supplementary Material online), which is a >3-fold overrepresentation relative to the expectation (see Results). Changes to the coding sequence of transcription factors could change their 3D structure, target binding site, specificity, and their ability to recruit other transcription factors. Most importantly, changes in transcription factors could lead to modified transcription levels of genes at many other loci in the genome, in contrast to protein-coding changes in structural or signaling proteins which only affect the protein in which they occur.

Organisms can adapt to new environments by regulating gene expression at multiple stages of mRNA biogenesis, a process governed by many different proteins, such as transcription factors, chromatin-remodeling factors, signaling molecules, and receptors (Kadonaga 2004; De Nadal et al. 2011). The second and the third most frequent protein class categories, transferases, and enzyme modulators, respectively, are also involved in gene expression regulation (fig. 7*B*). We checked all these protein class categories manually, and identified a total of 34 genes (out of the 39 genes that could be assigned to a protein class by PANTHER) that are involved in regulating gene expression (supplementary file 5, Supplementary Material online). That is, a majority of those genes (87%) are related to transcriptional regulation. Altogether, our results indicate that TRs in bumblebee drive potentially functional variability at loci involved in gene expression regulation and other biological functions. As a result, length variation of TRs may facilitate the adaptation of bumblebees through diversifying bumblebee proteins, particularly those which regulate gene expression, as has been previously hypothesized (King and Wilson 1975; Wray 2007; Fraser 2013).

## Conclusions

In this study, we performed a comprehensive investigation of TRs in the assembled genome of *B. terrestris*. We found out that TRs represent a significant source of genetic variation in bumblebees. We found TRs contribute to coding sequence variation and thereby likely influence the functional diversity of bumblebee genes. The functional roles of genes whose coding sequences contain variable-length TRs were analyzed, and our results indicate that a majority of those genes are related to transcriptional regulation. Given the importance of gene expression changes for adaptation, our observation that loci encoding transcription factors are enriched for variable-length TRs may suggest an important role for expanded repeats in the evolution of bumblebees. 

## Supplementary Material

Supplementary data are available at *Genome Biology and Evolution* online.

## Supplementary Material

Supplementary Data
