# Supplementary material for: Tandem Repeats Contribute to Coding Sequence Variation in Bumblebees (Hymenoptera: Apidae)
Source: Genome Biol Evol. 2018 Nov 6;10(12):3176–87. doi: 10.1093/gbe/evy244 (PMC6286909; doi:10.1093/gbe/evy244)
Supplement: Supplementary Data [file evy244_supp.zip › Legends supplementary materials.docx]

**Supplemental material Legends**

**Supplementary file 1.** This file contains detailed information for the PCR amplification of TR loci in coding sequences, which includes TR loci coordinates, primer sequences, PCR reaction conditions and amplification results etc.

**Supplementary file 2.** This file shows the pairwise alignments of the identified 1,137 variable-length TRs between *B. terrestris* and *B. impatiens*.

**Supplementary file 3.** This file shows the pairwise alignments of the identified 101 variable-length TRs in coding sequences between *B. terrestris* and *B. impatiens*.

**Supplementary file 4.** This file shows the pairwise alignments of proteins sequences encoded by genes containing variable-length TRs between *B. terrestris* and *B. impatiens*.

**Supplementary file 5.** This file shows the coordinates and repeat unit length for the 101 variable-length TRs in coding sequences. Proteins encoded by genes harboring those variable-length TRs and their corresponding protein class categories, if related to transcription regulation, are also shown.

Table 1 The summary for the PCR amplification of TR loci in coding sequences

| Total loci # | Successfully Amplified # | Fixed variation # | Variation within species # | Not fixed # |
| --- | --- | --- | --- | --- |
| 30 | 29 | 19 | 8 | 2 |

Note: detailed results are available in Supplemental file 1.
